# Smaller scale, same impact: replicating high-throughput phenotypic profiling in a medium-throughput lab for use in chemical risk assessment

**DOI:** 10.1007/s00204-025-04165-2

**Published:** 2025-08-28

**Authors:** Eunnara Cho, Stephen D. Baird, Kristin M. Eccles

**Affiliations:** 1 Environmental Health Science and Research Bureau, Healthy Environments and Consumer Safety Branch (HECSB) Health Canada, Ottawa, ON K1A 0K9 Canada; 2 High-Throughput Screening Lab, Children’s Hospital of Eastern Ontario Research Institute, Ottawa, ON K1H 8L1 Canada

**Keywords:** High-throughput phenotypic profiling, Cell Painting, High-content imaging, Toxicity assessment

## Abstract

Cell Painting visualizes toxicity-induced morphological changes by staining cellular structures with fluorescent dyes. Coupled with high-content imaging and analysis software, Cell Painting allows high-throughput phenotypic profiling (HTPP) to quantify phenotypic changes and estimate points of departure for toxicity assessments. Regulatory agencies have applied HTPP in 384-well plates for chemical hazard screening. In this study, established protocols for 384-well plates were adapted for use in 96-well plates to increase accessibility for laboratories with lower throughput. U-2 OS human osteosarcoma cells in 96-well plates were exposed to 12 phenotypic reference compounds for 24 h before fixation and staining with fluorescent dyes (golgi apparatus, endoplasmic reticulum, nucleic acids, cytoskeleton, mitochondria). Four independent chemical exposures across eight concentrations generated four biological replicates. Stained cells were imaged on an Opera Phenix, a high-content imaging system, and the Columbus analysis software extracted numerical values for 1300 morphological features. Features were normalized to control cells, followed by principal component analysis and a calculation of Mahalanobis for each treatment concentration. Mahalanobis distances were modeled to calculate benchmark concentrations (BMC) for chemicals. Most BMCs differed by less than one order of magnitude across experiments, demonstrating intra-laboratory consistency. Compared to published BMCs, ten compounds had comparable BMCs in both plate formats. In addition, we observed a significant inverse relationship between seeding density and Mahalanobis distances, suggesting that experimental factors like cell density may influence BMCs. Overall, we demonstrate that Cell Painting is adaptable across formats and laboratories, supporting efforts to develop and validate it as a complementary new approach methodology to existing toxicity tests.

**Supplementary Information:**

The online version contains supplementary material available at 10.1007/s00204-025-04165-2.

## Introduction

The omnipresence of chemicals in the environment and their potential hazards to human health necessitate comprehensive and robust toxicity assessment methods that can screen many chemicals rapidly (Richard et al. 2021). Traditional toxicological approaches often rely on animal testing and a limited number of biological endpoints, which are increasingly recognized as insufficient to capture the complex and multifaceted nature of chemical exposure hazards (Kavlock et al. 2009; Krewski et al. 2020). As a result, there has been a focus on developing and implementing innovative, high-throughput methods for toxicity testing that, individually or in conjunction, can provide a more holistic and mechanistic understanding of chemical toxicity (Krewski et al. 2020). Among these emerging techniques, Cell Painting, a high-throughput phenotypic profiling (HTPP) method, has gained considerable attention for its potential to revolutionize hazard assessment of environmental chemicals (Bray et al. 2016; Nyffeler et al. 2020; Willis et al. 2020; Cimini et al. 2023; Seal et al. 2025).

Cell Painting uses multiplexed fluorescence microscopy to capture a large number of cellular features, such as organelle morphology, following chemical perturbations (Gustafsdottir et al. 2013). This method generates large, high-dimensional datasets that can be quantitatively analyzed using computational methods to reveal phenotypic changes indicative of cellular perturbations caused by chemical exposure. When coupled with computational workflows and statistical methods such as multivariate analysis techniques, phenotypes can be associated with toxicity, and concentration–response modeling can be used to determine points of departure (POD) of chemical-induced phenotypic changes for use within quantitative chemical hazard assessment. The high-throughput nature of Cell Painting enables the screening of large chemical libraries across multiple cell types, making it a powerful tool for identifying chemical hazards and elucidating their mechanisms of action.

Cell Painting has predominantly been used in the drug discovery space (Swinney and Anthony 2011; Moffat et al. 2014; Li and Xia 2019; Fredin Haslum et al. 2024) and, more recently, for hazard screening of environmental chemicals. The United States Environmental Protection Agency (EPA) has used a LabCyte Echo 550 acoustic dispenser for a randomized chemical dosing in a 384-well plate and an Opera Phenix High-Content Screening System (PerkinElmer) for image acquisition for over 1200 chemical exposures (Nyffeler et al. 2023) or in a variety of different cell lines (e.g., U-2 OS, MCF7, HepG2, A549, HTB-9, ARPE-19 (Willis et al. 2020), and hNP1 (Culbreth et al. 2022)). Their work showed a good concordance between the bioactivity predictions (i.e., benchmark concentrations (BMC)) across different cell lines (Willis et al. 2020), and the corresponding administered equivalent dose (AED) for each bioactivity was as conservative or even more protective than the comparable in vivo effect levels 68% of the time. This demonstrates that HTPP is a cost-effective and efficient method for screening hazards related to exposure to environmental chemicals (Nyffeler et al. 2020). When HTPP data were combined with other toxicity endpoints, such as transcriptomic responses, the two streams of evidence collectively provided complementary but unique data (Nassiri and McCall 2018; Nyffeler et al. 2022; Way et al. 2022; Bundy et al. 2024), and these methods produced similar potency rankings (within an order of magnitude) (Nyffeler et al. 2022).

The use of HTPP represents a significant advancement in the field of chemical hazard assessment by adding another line of evidence to the toolkit of high-throughput screening methods, including cell-based assays and transcriptomics (Nyffeler et al. 2023; Bundy et al. 2024). The application of Cell Painting in conjunction with transcriptomics aligns with the Adverse Outcome Pathway (AOP) framework, which links molecular initiating events to adverse health outcomes through a series of biological key events (Ankley et al. 2009). By providing detailed phenotypic and molecular data, this combined approach can enhance the mechanistic understanding of chemical toxicity and support the construction and validation of AOPs, which can inform regulatory decision-making.

While large-scale facilities with access to high-throughput technologies can generate large datasets quickly, many research laboratories operate with more limited resources and infrastructure. Thus, building confidence in phenotypic profiling in medium-throughput laboratories using 96-well plates is crucial for broadening the accessibility and utility of these advanced methodologies in laboratories without automated liquid handling capabilities. Svenningsen and Poulsen (2019) detailed a protocol that established Cell Painting in medium-throughput laboratories, citing that the experimental methods have been well-described (Bray et al. 2016; Cimini et al. 2023), and it was the computational components, including data handling, analysis, and programming requirements, that were the biggest hurdles (Svenningsen and Poulsen 2019).

Herein, we aimed to replicate the EPA’s HTPP results in U-2 OS human osteosarcoma cells (Nyffeler et al. 2020) in an independent, medium-throughput laboratory. We adapted the Cell Painting protocols used for 384-well plates described by Bray et al. (2016) and Cimini et al. (2023) to allow for assaying in a 96-well plate. Promoting smaller scale Cell Painting in medium-throughput laboratories will enable a broader application of HTPP in hazard assessment and a more widespread adoption and standardization of HTPP as a toxicity assay. A greater diversity of chemicals and cell lines can then be assessed across various conditions, contributing to a more comprehensive understanding of toxicity and the risks associated with exposure to environmental chemicals. Furthermore, demonstrating inter-laboratory reproducibility is necessary to build confidence in the robustness of the Cell Painting methodology as an alternative method for toxicity testing and to gain acceptance within the scientific community and regulatory space.

## Materials and methods

### Cell culture

U-2 OS human osteosarcoma cells (ATCC, cat# HTB-96, Manassas, VA) frozen at P4 (2 × 10^6^ cells/vial) were thawed prior to each experiment and were exposed to chemicals within three passages. Cells were cultured in a T75 flask (Corning) in 20 mL of McCoy’s modified 5a medium (ATCC; 30–2007), as per ATCC’s recommendation, supplemented with 10% v/v fetal bovine serum (Gibco) and 1% penicillin–streptomycin (Gibco) at 37 ℃ in 5% CO_2_. We note that this is different from the Nyffeler et al. (2020) study, which used Dulbecco’s modified Eagle medium (DMEM). Cell density was maintained below 80–90% confluence. To passage the cells, cells were washed in 1 × phosphate-buffered saline solution prior to trypsinization with 0.25% trypsin (Gibco) for up to 5 min at 37 ℃. Cells were suspended in 9 × volume of the maintenance medium to deactivate trypsin. An aliquot (~ 100 µL) of the suspension was collected for Trypan blue staining and counting using an automated cell counter (Countess 3, Invitrogen) and seeded in a T75 flask (maintaining 1 × 10^5^ – 1 × 10^6^ cells/mL) or in 96-well plates (PhenoPlate 96-well microplates, Revvity) 24 h prior to chemical exposures. In 96-well plates, cells were seeded at a density of 5000 cells/well in 100 µL of media using a manual 12-channel pipette.

### Chemical exposures

HTPP reference compounds (Nyffeler et al. 2020) (Table 1) were dissolved in cell culture grade dimethyl sulfoxide (DMSO) (ATCC) to prepare stock solutions at varying concentrations. Treatment solutions were prepared in sterile 96-well plates at 200 × treatment concentration in DMSO. Following the top concentration, the subsequent seven concentrations were spaced by a half-log unit. In a sterile deep-well 96-well plate, 350 µL of exposure media was prepared for each concentration of each reference chemical by adding the treatment solutions to the media at 0.5% v/v. Vehicle controls were prepared with DMSO at a concentration of 0.5% v/v in the media. To expose the cells, the media in the 96-well plate containing the cells was removed and replaced with exposure media prepared in deep-well plates using a 12-channel pipette. Cells were exposed for 24 h. Exposures were performed four times in four independent experiments, where P4 vials of U-2 OS cells were thawed and cultured for each experiment. Each experiment included four 96-well plates, each containing eight DMSO-treated wells (vehicle control), eight concentrations of sorbitol (phenotypic negative control), eight concentrations of staurosporine (cytotoxic control), and eight concentrations of three test chemicals in triplicate. Supplementary Fig. 1 illustrates the plate layout. Well positions were arranged in a fixed order rather than randomized. The highest concentration of each test chemical was placed in row A, with the subsequent concentrations in rows B to H in a decreasing order. Replicates of each concentration were placed in adjacent wells. Experiments 1–3 were identical, containing all 14 chemicals listed in Table 1. Experiment 4 contained amperozide, berberine chloride, fluphenazine, NPPB, and tetrandrine, in addition to the two controls, staurosporine and sorbitol.

**Table 1:** Summary of reference compounds used in this validation study

Chemical	CAS	Vendor	Cat. No.	Target (Gustafsdottir et al. 2013)	Stock (mM)	Tested Concentration range (µM)
5-Nitro-2-(3-phenylpropylamino)benzoic acid	107,254–86-4	Caymen Chemicals	17,292	Redistribution of ER to one side	20	100–0.1
Amperozide	86,725–37-3	MedChemExpress	HY-121301	Toroid nuclei	20	100–0.1
Berberine Chloride	633–65-8	Millipore Sigma	B3251	Redistribution of mitochondria	20	100–0.1
Ca-074-Me	147,859–80-1	Millipore Sigma	C5857	Bright, abundant Golgi staining	20	10–0.01
Etoposide	33,419–42-0	Sigma	E1383	Large, flat nucleoli	20	10–0.01
Fenbendazole	43,210–67-9	Toronto Research Chemicals	TRC-F246750	Cytotoxic control	20	2–0.002
Fluphenazine	69–23-8	Toronto Research Chemicals	TRC-F598418	Giant, multi-nucleated cells	20	20–0.02*
Latrunculin B	76,343–94-7	Millipore Sigma	428,020	Actin breaks	20	10–0.01
Metoclopramide	364–62-5	Millipore Sigma	32,473	Enhanced Golgi staining and fused nucleoli	50	100–0.1
Taxol (paclitaxel)	33,069–62-4	StressMarq Biosciences	SIH-239	Large, multi-nucleated cells with fused nucleoli	20	11–0.003*
Rapamycin	53,123–88-9	Caymen Chemicals	13,346	Reduced nucleolar size	20	20–0.02*
Tetrandrine	518–34-3	Millipore Sigma	SML3048	Abundant ER	20	100–0.1
Sorbitol	50–70-4	Millipore Sigma	S1876	Negative control	20	25–0.025
Staurosporine	62,996–74-1	Millipore Sigma	S5921	Cytotoxic control	20	0.5–0.0004

All chemicals were dissolved in DMSO.

The concentration ranges were determined based on Nyffeler et al. (2020) with minor changes (*):

The top concentrations of fluphenazine and taxol were reduced from 100 µM to 20 and 11 µM, respectively. The top concentration of rapamycin was increased to 20 from 10 µM.

In addition, one 96-well plate of cells was treated with 0.5% v/v DMSO only to examine variations in cell count and background variability in phenotypic changes with exposure to DMSO across the wells.

### Cell staining

Liquid handling in 96-well plates was performed manually using a 12-channel pipette unless otherwise specified. U-2 OS cells were fixed in paraformaldehyde and stained per the protocol described by Cimini et al. (2023). The volumes were adjusted for 96-well plates. The concentrations of the fluorescent dyes in the wells were consistent with those reported by Cimini et al. (2023). These concentrations differ from the concentrations used by Nyffeler et al. (2020), which were higher for all dyes, except for MitoTracker Red. We conducted pilot experiments (data not shown) using the Cimini et al. (2023) concentrations to conserve dyes and retained these concentrations for the definitive study after confirming the high quality of the resulting images.

Briefly, all dyes were solubilized in their respective solvents to prepare stock solutions at concentrations specified in Table 2. After 24 h of chemical exposure, approximately 50% of the exposure media (45 µL) was removed from each well and replaced with 27 µL of MitoTracker Red working solution (final concentration of 0.5 µM in the well) and the cells were incubated at 37 °C for 30 min. Cells were fixed by adding 27 µL of 16% paraformaldehyde aqueous solution (Electron Microscopy Sciences; cat# 15,710) to each well (approximately 4% final concentration in the well) and incubating at room temperature for 20 min in the dark. Using an automated plate washer (BioTek EL406, Agilent), all liquid in the wells was aspirated, and cells were washed four times with 100 µL of 1 × Hank’s Balanced Salt Solution (HBSS; Gibco).

**Table 2:** Fluorescent dyes and concentrations used for Cell Painting

Dye	Staining solution	Invitrogen product #	Solvent	Dye stock concentration	Dye working concentration in well (Cimini et al. 2023)	Dilution factor in well	Storage temperature
MitoTracker Red	Live	M22426	DMSO	1 mM	500 nM	2000	−20 °C
Alexa Fluor™ 568 Phalloidin	Fixed	A12380	MeOH	6.6 µM	8.25 nM	800	−20 °C
Alexa Fluor™488 Concanavalin A	Fixed	C11252	0.1 M NaHCO_3_	2 mg/mL	5 µg/mL	400	−20 °C
SYTO14	Fixed	S7576	N/A	5 mM	6 µM	833	−20 °C
Alexa Fluor™ 555 Wheat Germ Agglutinin	Fixed	W32464	dH_2_O	1 mg/mL	1.5 µg/mL	667	−20 °C
Hoechst 33,342	Fixed	H3570	dH_2_O	10 mg/mL	1 µg/mL (162 nM)	10,000	4 °C

Fixed cells were permeabilized and stained in 40 µL of the dye working solution (1 × HBSS with 1% w/v bovine serum albumin, 0.1% v/v Triton X-100, 8.25 nM Alexa Fluor 568 Phalloidin, 5 µg/ml Alexa Fluor 488 Concanavalin A, 1 µg/ml Hoechst 33,342, 6 µM SYTO 14, and 1.5 µg/ml Alexa Fluor 555 wheat germ agglutinin). After a 30-min incubation in the dark at room temperature, using the plate washer, the staining solution was aspirated from all wells, and the wells were washed four times with 100 µL of 1 × HBSS, with the last portion of HBSS retained in the wells for storage of the plate and imaging. The plates were stored in the dark at 4 °C until imaging. The plates were imaged within a week of staining.

### Image acquisition

Stained cells in 96-well PhenoPlates were imaged using an Opera Phenix High-Content Screening System (Revvity) as described by Nyffeler et al. (2020), with minor changes. Each well was imaged four times at differing fluorescence excitation wavelengths: 375 nm (Hoechst 33,342: DNA), 488 nm (Alexa Fluor 488 Concanavalin A: ER; SYTO14: Nucleoli and RNA), 561 nm (Alexa Fluor 568 Phalloidin: actin; Alex Fluor 555 Wheat germ agglutinin: Golgi apparatus, plasma membrane), and 640 nm (MitroTracker Red: mitochondria). The 488 nm channel was 1 µm above the 375 nm, 561 nm, and 647 nm channels. A 20 × water objective with 2 × 2 pixel binning was used, consistent with Nyffeler et al. (2020). Slight changes were made from the Nyffeler et al. (2020) procedure, where the exposure was set by having positive signals of each channel (approximately 10–20 × signal to background noise) in the relative range of 3000 Intensity units, as recommended by Revvity, the manufacturer of the Opera Phenix system.

In pilot experiments, we observed variabilities in cell density within each well, with certain areas having higher density than others. In addition, the location of high-density areas varied across different wells on the plate. We addressed this issue by distributing the fields of view across the well rather than imaging just the center of the well. Thus, nine different fields across the well were imaged within each well with a combined area of 9,720 µm × 9,720 µm, instead of imaging nine neighboring fields of view in the center of the well (Supplementary Fig. 2). Although the number of cells in each field of view was variable, this imaging method resulted in a more consistent median number of cells imaged in each vehicle control-treated well across the plate.

### Image processing

Images were processed using the Columbus Scope image management and analysis software (Revvity, formerly PerkinElmer), as described by Nyffeler et al. (2020). Briefly, nuclei were identified and segmented in the 375-nm channel (DNA) and were used as a guide to segment whole cells in the 488-nm channel (ER/nucleoli/RNA). Cells were filtered for analysis based on cell area (> 100 µm^2^ and < 6700 µm^2^), nucleus area (< 1000 µm^2^), nucleus roundness (> 0.5), and the intensity in the 488-nm channel (> 500). Cells on image borders were excluded. Next, cells were further segmented into the membrane (5 pixels inward from the cell’s outer boundary), ring (50% of the distance from the nucleus to the cell’s outer edge), and cytoplasmic regions (5 pixels from the cell’s outer edge to the nucleus). In each of the defined regions (cell, nucleus, cytoplasm, membrane, ring), different features were measured using the Columbus software. Features such as intensity, texture, SCARP (symmetry, compactness, axial, radial, and profile) morphology, and basic morphology (e.g., area, roundness, width, length, width-to-length ratio) were measured, with multiple properties contained within each feature category. In total, 1300 features were measured in each cell. The features can be grouped into 49 categories.

### Computational pipeline

The numeric cell-level data from Columbus were processed in the R programming environment (R Core Team 2024). While we used the same normalization and data reduction methods as Nyffeler et al. (2020, 2021), our R data processing pipeline (https://github.com/HC-EHSRB-CompTox/Cell-Painting-Pipeline) was modified to accommodate our 96-well experimental design, which is explained herein.

Raw data from four plates in each experiment were merged and treated as one batch for analysis. The total cell count in each well was determined to calculate the percent reduction in cell count in reference compound-treated wells relative to the median cell count of the vehicle control wells. Wells with fewer than 100 analyzable cells or those exhibiting greater than 50% reduction in cell count were excluded. The median and median absolute deviation (MAD) of all feature values of analyzable cells in all DMSO wells in the batch were calculated. All raw feature values of each cell in the reference compound-treated wells were normalized to the DMSO wells by subtracting the DMSO median and dividing by the DMSO MAD. The cell-level values were aggregated to the well level by calculating the well median of each feature. The standard deviation (SD) of the DMSO wells across the batch was calculated. The well-level medians of the treated wells were further normalized by dividing by the SD of the DMSO wells (normalized well-level feature data are available in the Supplementary Materials).

The normalized well-level feature values of each batch were reduced to one set of principal components (PC) for global concentration–response modeling or 49 sets of PCs for categorical concentration–response modeling by conducting the principal component analysis (PCA) using the R function *prcomp* (center = T and scale = T) in R *stats* package (v 4.4.1) (R Core Team 2024). For the categorical analysis, the dataset was divided into 49 subsets, each containing only features that belong in the category and treated as an independent dataset.

Following the PCA, a subset of PCs that described 95% of the variance in the dataset was identified. We calculated the covariance matrix of this subset using the *cov* function (*stats* package). To calculate the Mahalanobis distance of each well from the mean of the DMSO-treated wells, the covariance matrix was first inverted using the *ginv* function (*MASS* package: v7.3–64) (Venables and Ripley 2002). The inverted matrix was input in the *mahalanobis* function (*stats* package) along with the PCs of the dataset and the center defined as the mean PCs of DMSO wells, to calculate the global or categorical Mahalanobis distances of each well.

The *tcplfit2* R package (Sheffield et al. 2022) was used to model concentration–response in the Mahalanobis distance in response to the phenotypic reference chemicals. As per the method used by Nyffeler et al. (2021), the benchmark response (BMR) for deriving the BMC was set as one MAD above the median Mahalanobis distance of the DMSO wells in the batch. The concentration–response of the Mahalanobis distance relative to the control compound (DMSO) was fitted to multiple models. The fit with the lowest Akaike Information Criterion (AIC) was selected as the winning model for each chemical. BMCs with a hitcall below 0.9 were excluded, as were BMCs that were above the highest tested concentration or without an upper or lower confidence limit.

Our pipeline was validated by processing publicly available well-level feature values from Nyffeler et al. (2020) (24-h exposures in U-2 OS cells in 51 384-well plates; URL: https://gaftp.epa.gov/COMPTOX/CCTE_Publication_Data/BCTD_Publication_Data/Nyffeler/) and comparing the BMCs produced using our pipeline against the published values in the 2020 and 2021 studies. The first 3 of the 51 published plates contained 14 phenotypic reference compounds only, and the remaining 48 plates included test chemicals with berberine chloride, Ca-074-Me, etoposide, and rapamycin as phenotypic positive controls.

### Analysis of DMSO-treated 96-well plate

To assess the impact of solvent control position on Mahalanobis distance calculations, the 96-well plate of U-2 OS cells treated only with 0.5% DMSO was normalized to the median of all wells or individual rows and columns. Each of the 12 columns and 8 rows were systematically designated as the solvent control in separate iterations, resulting in 20 total normalizations. Mahalanobis distances were recalculated for each set of normalized well-level feature values to evaluate the effect of control position across the plate.

Linear regression of Mahalanobis distance against cell count per well was performed using the lm() function from the R *stats* package. The strength of the association between these parameters was evaluated using the adjusted R-squared value.

## Results

### Validation of our computational pipeline

**Fig. 1:**
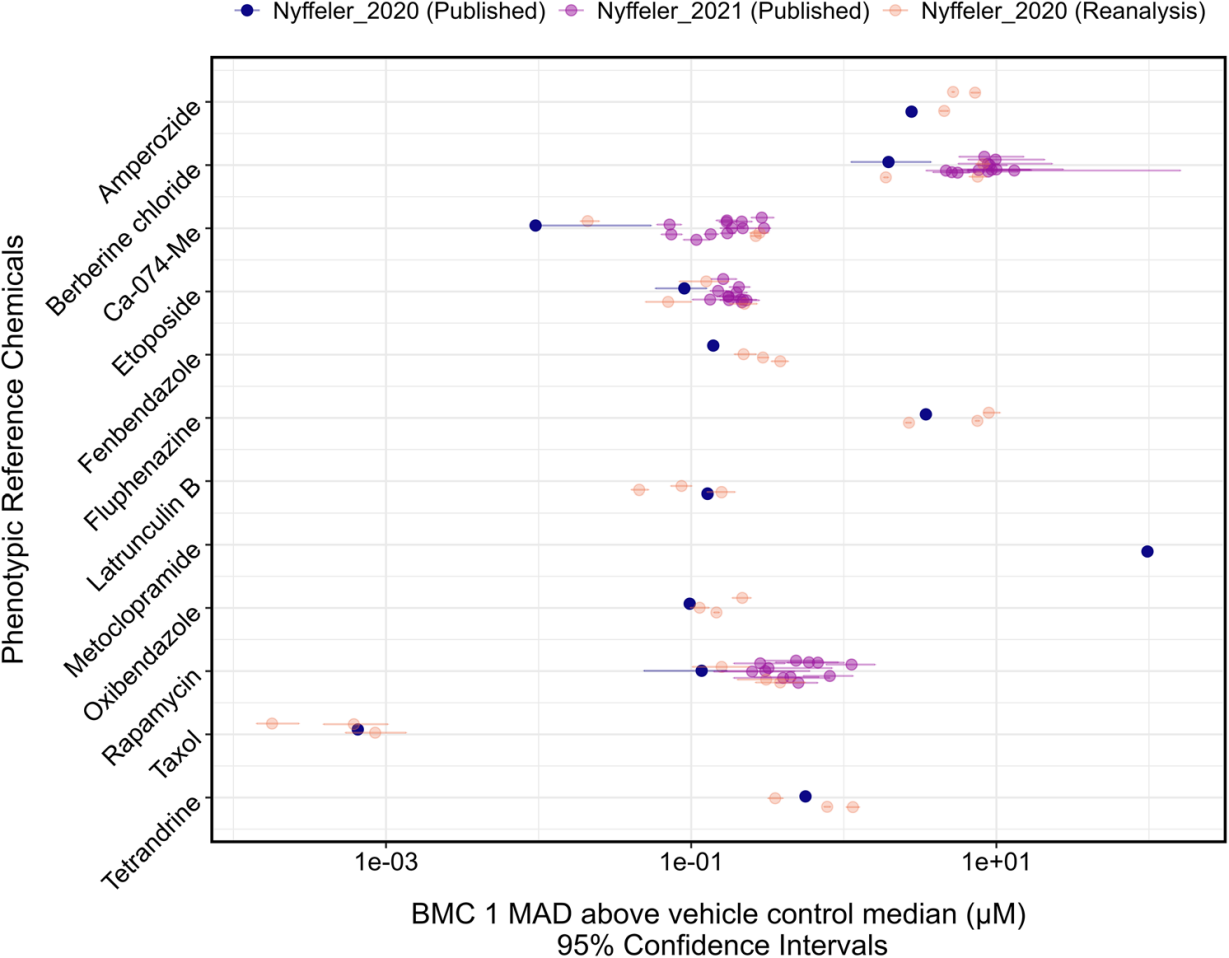
Comparison of benchmark concentrations (BMCs) for phenotypic reference compounds calculated from the well-level data generated in the Nyffeler et al. (2020) study. BMCs were calculated using our modified computational pipeline and compared to the values originally reported in Nyffeler et al. (2020, 2021). Bars represent the 95% confidence intervals (CIs) of the BMCs, defined as the concentrations at which the Mahalanobis distance exceeded one median absolute deviation (MAD) above the vehicle control median

To validate the performance of our modified pipeline, we reanalyzed three 384-well plates containing 14 phenotypic reference compounds from the publicly available well-level dataset for 51 384-well plates imaged in the study by Nyffeler et al. (2020). The BMC values for a BMR of one MAD above the vehicle control median computed using our pipeline were compared against the reported values in Nyffeler et al. (2020, 2021) (Fig. 1).

In Nyffeler et al. (2020), dimensionality reduction (PCA) was not performed, and curve-fitting was conducted for individual features using normalized well-level values in BMDExpress, a software for concentration–response modeling and BMC calculations. The features were grouped into 49 categories, and the median BMC for each category was calculated. The lowest BMC, thus, the most sensitive feature category, was reported as the overall point of departure (POD) for the chemical (Nyffeler et al. 2020). Since confidence intervals were not reported, we estimated the 95% confidence intervals (CIs) using the 5th and 95th quantiles. In the 2021 study, the 2020 dataset was reanalyzed using dimensionality reduction by PCA and calculating the Mahalanobis distances, and the *tcplfit2* package was used to model the global concentration–response and calculate the global BMCs for a BMR of one MAD above control median for each chemical (Nyffeler et al. 2021). The data from four 384-well plates from four individual U2-OS cultures treated with the same 384-well “dose plate” (a plate containing the test chemicals and dilutions) were combined and collectively treated as one replicate. Twelve BMC values were reported for each of berberine chloride, Ca-074-Me, etoposide, and rapamycin in the 2021 publication (Fig. 1).

In the reanalysis of the Nyffeler et al. (2020) data using our modified pipeline, 12 of the 14 phenotypic reference compounds had BMCs within one order of magnitude of the 2020 BMCs. The median BMCs of each chemical from the three analyses differed by less than one order of magnitude (ranging from 0.002 to 0.65), except for Ca-074-Me, whose median BMC from the current analysis differed by one order of magnitude from the median BMC of the published 2020 study. No BMCs were calculated for NPPB in any of the three analyses. A BMC for metoclopramide was produced in the 2020 analysis only. The 2021 study reported 12 BMCs for 4 reference compounds: berberine chloride, etoposide, rapamycin, and Ca-074-Me (Nyffeler et al. 2021). All four compounds had BMCs from our pipeline that were less than one order of magnitude different from the published BMCs. However, these BMCs reported within the 2021 paper, at times, spanned more than one order of magnitude, though they generally encompassed the published values in 2020. Notably, Ca-074-Me had a larger difference between the 2020 and 2021 published values. The BMC result from our computational pipeline fell between these two reported results. There was no overarching trend as to whether the BMCs from our pipeline were higher or lower than the published result.

### BMC variability between experiments in 96-well plates

**Fig. 2:**
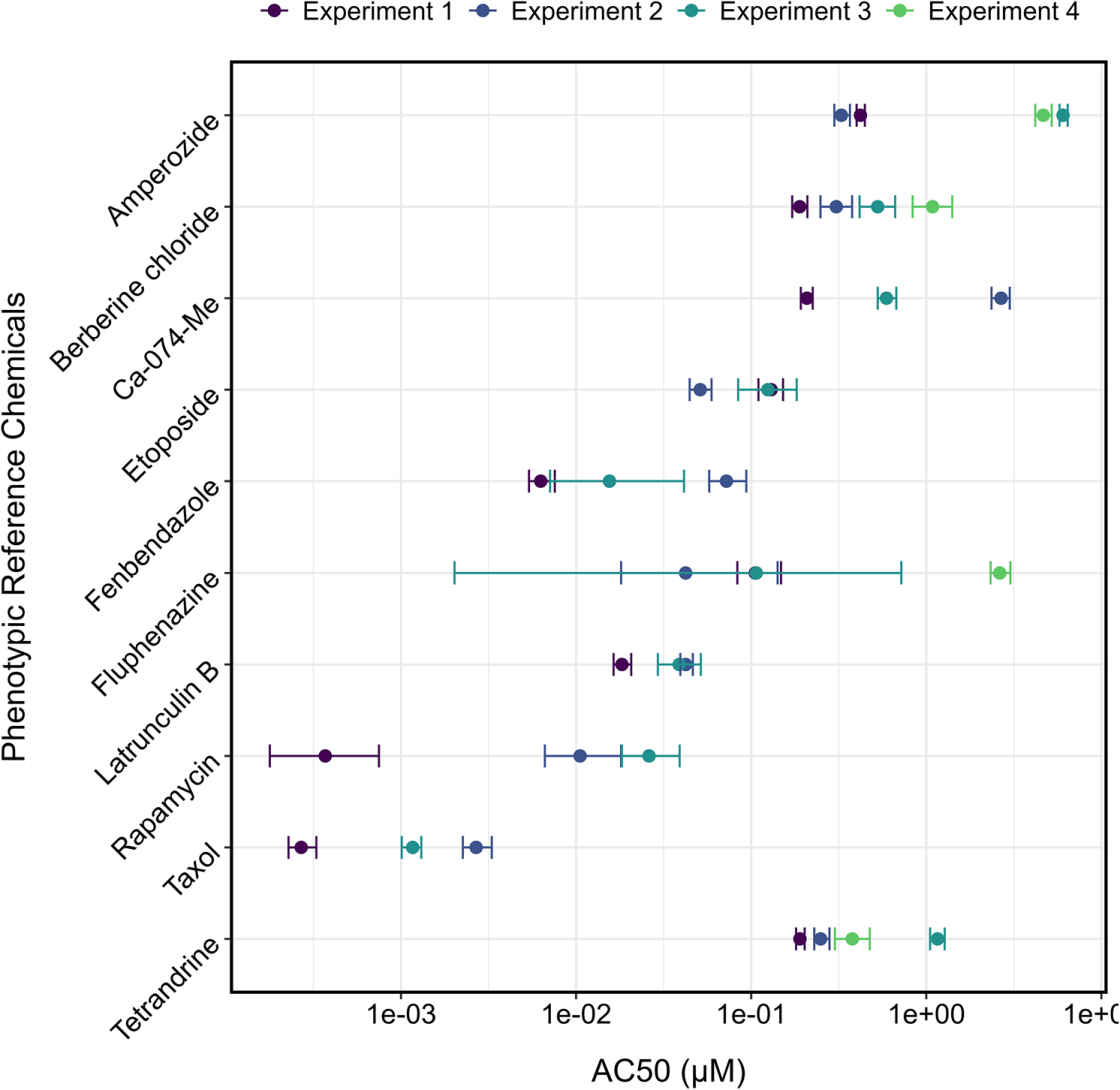
Comparison of the BMCs of the phenotypic reference compounds calculated in four independent experiments. The bars represent the 95% confidence intervals (CI) of the BMC values. The BMCs indicate the concentrations at which there was an increase in Mahalanobis distance, one median absolute deviation (MAD) above the vehicle control median

We exposed our U-2 OS cells to 12 phenotypic reference compounds for 24 h in 96-well plates, and the experiment was repeated in four independent trials. Figure 2 summarizes the BMCs that met our inclusion criteria (hitcall > 0.9; defined upper and lower confidence limits) from the four experiments. Metoclopramide was excluded from Fig. 2 because it did not produce any BMCs that met the criteria. Only one experiment yielded a BMC for NPPB that met the criteria. This is consistent with the results in the Nyffeler et al. (2020 and 2021) studies.

Many BMCs were less than one order of magnitude different across the experiments for 10 of the 12 tested chemicals, with many of the 95% confidence intervals overlapping between experiments. For five chemicals (amperozide, berberine chloride, Ca-074-Me, taxol, tetrandrine), none of the CIs overlapped between the experiments. Further, for chemicals including rapamycin and fluphenazine, some experiments had large confidence intervals, with the repeat experiments spanning more than one order of magnitude. The four reference compounds selected by the EPA, berberine chloride, etoposide, rapamycin, and Ca-074-Me, for use as phenotypic positive controls on plates with test chemicals, for the most part, were close together, having overlapping confidence intervals or two or more replicates within an order of magnitude, indicating that they are reproducible across experiments.

### Comparison of cell painting benchmark concentrations against published values

**Fig. 3:**
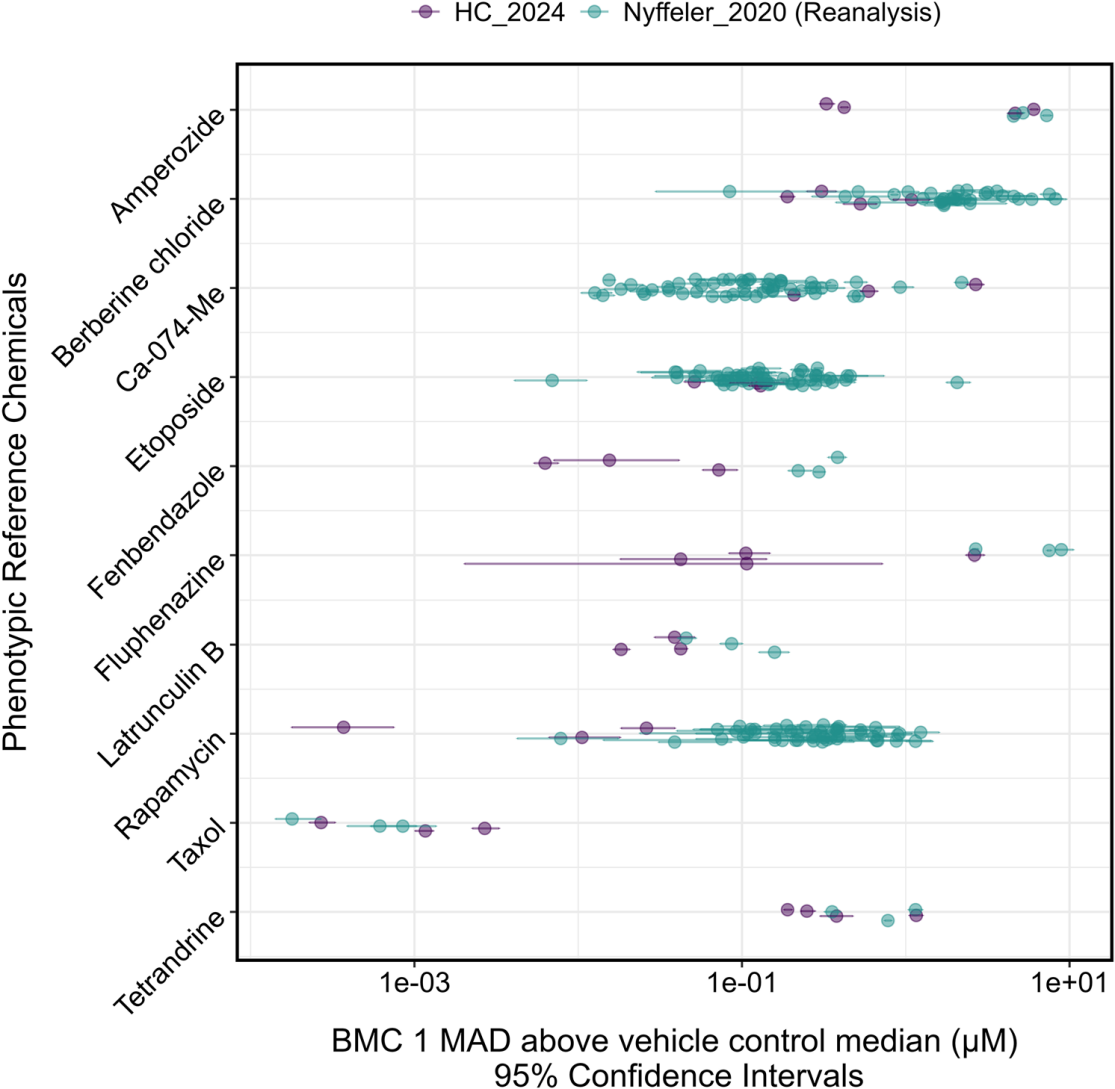
Comparison of the benchmark concentrations (BMCs) of the phenotypic reference compounds calculated in the four independent experiments in this study (HC) and the BMCs calculated from the Nyffeler et al. 2020 dataset. The Nyffeler et al. (2020) dataset contained 51, 384-well plates with 3 plates containing only the reference compounds and the remaining 48 plates containing test chemicals for hazard screening along with berberine chloride, Ca-074-Me, etoposide, and rapamycin as phenotypic positive controls. Each data point represents a BMC determined from one 384-well plate. The bars represent the 95% confidence intervals (CI) of the BMC values

To compare the Cell Painting results in 96-well plates against 384-well plates, we reanalyzed all 51 384-well plates in the Nyffeler et al. (2020) dataset and calculated the BMCs for the 12 phenotypic reference compounds shared between this dataset and the current study (Fig. 3). Two compounds, metoclopramide and NPPB, did not produce a positive response in one or both of the studies and, thus, were excluded from Fig. 3.

The 96-well plate BMCs were generally lower for many of the chemicals than those calculated from the 384-well plates. All ten chemicals that produced a phenotypic response had BMCs that were within one magnitude of each other or had one or more overlapping confidence intervals between the two studies and plate formats. Four reference compounds (rapamycin, berberine chloride, etoposide, and Ca-074-Me) that were included in all fifty-one plates in Nyffeler et al. (2020) had BMCs and CIs that roughly spanned an order of magnitude. The 96-well plate-calculated BMCs of etoposide, berberine chloride, and rapamycin were on the lower end of this range, while Ca-074-Me was on the higher end.

### Background variability of Mahalanobis distance

**Fig. 4:**
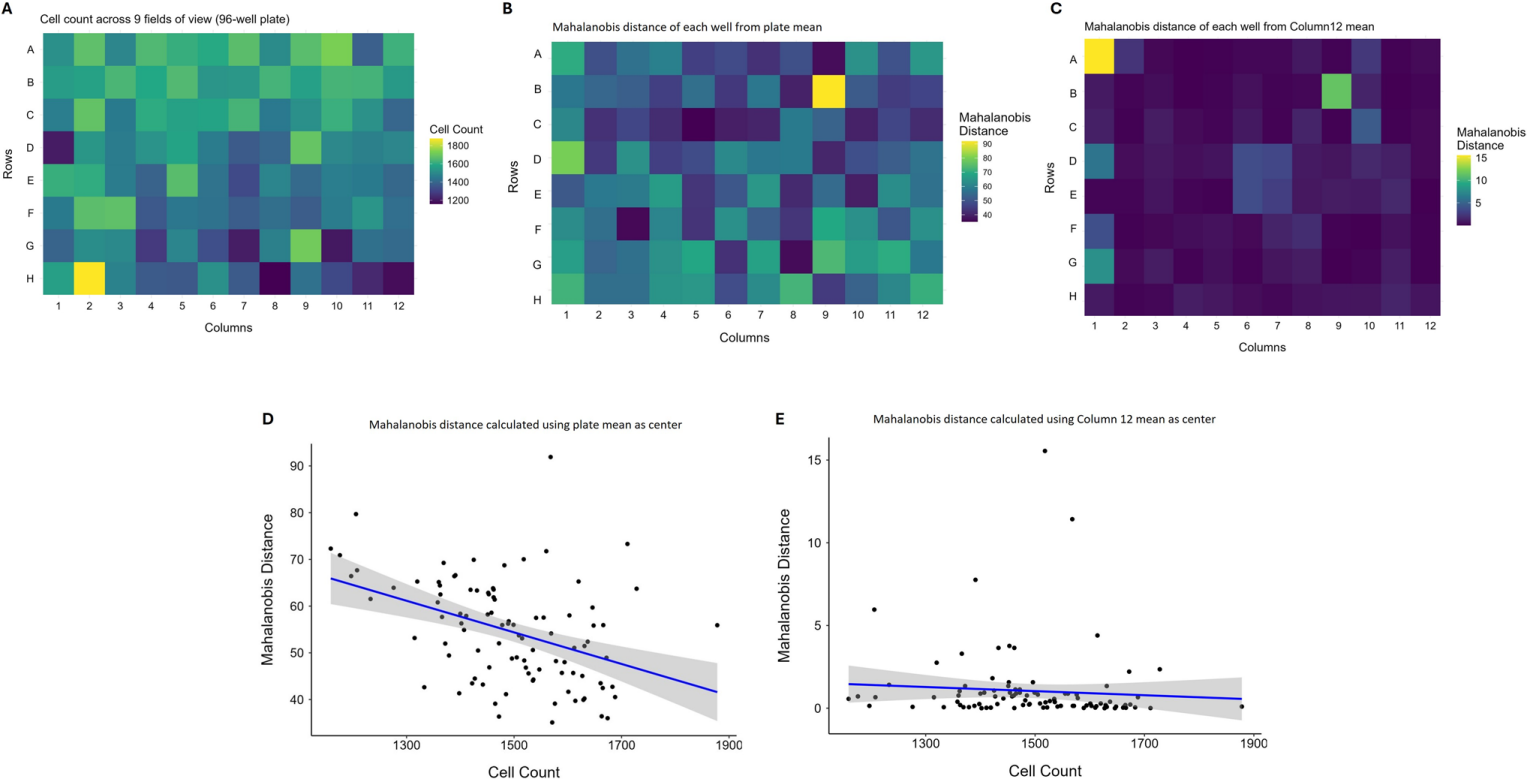
Impact of cell count variability on Mahalanobis distance under whole-plate and column-based reference strategies in a 96-well plate. One 96-well plate was treated with only DMSO (0.5% v/v in media), and the Mahalanobis distance from the plate mean and the column 12 mean was calculated for each well. Heatmaps of cell count in each well (**A**), Mahalanobis distance of each well from the plate mean (**B**), and from the column 12 mean (**C**). Linear regression analysis of cell count and Mahalanobis distance calculated from the plate mean (**D**) and the column 12 mean (**E**). There was a statistically significant effect of cell count on the Mahalanobis distance (*p* value < 0.001) of the wells from the plate mean

Mahalanobis distances were calculated for each well of a 96-well plate treated only with 0.5% v/v DMSO in media to observe background variability that occurs as a technical artifact (e.g., cell count/density) and its influence on the Mahalanobis distances. The mean of the entire plate was used as the center from which the distances were calculated. Overall, there was a decreasing trend in cell count from rows A to H, which was observable in the heatmap representing the cell count in each well of the plate, with the wells in row A generally having a higher cell count than the subsequent rows (Fig. 4A). The Mahalanobis distances had an opposite trend, generally smaller in the wells in row A, and increasing with subsequent rows (Fig. 4B). This suggests that cell count may impact the Mahalanobis distance, an observation that is supported by linear regression modeling using the cell counts to predict the Mahalanobis distances which generated a statistically significant negative relationship (t-value = − 4.41, *p* value < 0.001) (Fig. 4D). The adjusted R-squared indicated that 16.3% of the variance in the Mahalanobis distance could be attributed to the variance in cell count.

The Mahalanobis distances calculated with row- or column-based controls exhibited more consistency across the plate (Fig. 4C; Column 12 used as control). Linear regression models predicting Mahalanobis distance from cell count showed a weaker and less consistent relationship (*t* values ranging from − 0.78 to − 6.99) (Fig. 4E). The adjusted R-squared values indicated that cell count explained 0.1% to 11% of the variance in the Mahalanobis distance, with lower variance explained when using localized controls compared to whole-plate normalization. When linear regression was performed on the combined Mahalanobis distances calculated in all 20 iterations, the adjusted R-squared value indicated that less than 1% of the variance in Mahalanobis distance was explained by variability in cell count (Supplementary Fig. 5). This suggests that Mahalanobis distance is influenced by cell count, but the extent of this dependence is reduced when reference groups are spatially localized.

## Discussion

We used the established HTPP protocols for 384-well plates (Bray et al. 2016; Nyffeler et al. 2020; Cimini et al. 2023) and adapted the computational pipeline to be compatible with 96-well plates to facilitate the application of Cell Painting in laboratories without high-throughput liquid handling capabilities. The phenotypic BMC values from the Nyffeler et al. (2020, 2021) studies conducted in 384-well plates and those generated in this study were generally comparable, demonstrating that HTPP results are reproducible between the two laboratories and across two different plate formats. The results support the transferability of Cell Painting assay to medium-throughput laboratories, increasing the accessibility of this assay for chemical hazard assessments.

In addition to comparing against published results, we examined the intra-laboratory consistency of the BMC values by performing four independent experiments. While 10 of the 12 reference chemicals had BMCs within one order of magnitude of each other or overlapping 95% confidence intervals between two or more experiments, there were also one or more BMCs of every chemical that were significantly different between the experiments, indicating there was some variability between the experiments. We examined experimental factors that may influence the Mahalanobis distance to identify potential sources of plate-to-plate and across-experiment variabilities that could have led to the observed discrepancies in the BMCs.

We explored background variability in Mahalanobis calculations by exposing an entire 96-well plate of cells to only DMSO (0.5% v/v), given that BMCs are calculated using the baseline Mahalanobis distances of the vehicle controls as a reference point. When using the plate mean as the reference, we observed a significant inverse relationship between cell count and Mahalanobis distance, suggesting that wells with lower cell counts were more likely to be identified as phenotypic outliers (having a larger Mahalanobis distance). However, when we used an experimentally consistent reference group (e.g., column 12—the position of our solvent control), this relationship became less pronounced, with greater variability attributable to the choice of control well location rather than cell count alone. This suggests that the influence of cell count on Mahalanobis distance is, at least in part, determined by how the reference distribution is defined. Since individual rows or columns may not fully represent the spatial heterogeneity across a plate, these findings emphasize the importance of a consistent control placement or normalization strategy to minimize technical bias (e.g., from manual pipetting), particularly in smaller scale assays where even subtle positional effects can significantly impact results. Cell morphology is directly influenced by cell density; cell-to-cell contact modulates the localization and expression of protein markers of different cellular compartments (Wada et al. 2011; Yao et al. 2019). In addition, the density of cells within a well are heterogeneously distributed, and higher density areas within the wells contribute a larger number of cells to the well-level summary metrics (e.g., median, MAD) that are used to normalize cell-level values prior to well-level aggregation and PCA. Thus, the center (the mean PCs of the plate) from which the Mahalanobis distances are calculated may be skewed toward the wells with higher cell count and density, leading to smaller Mahalanobis distances in these wells. This may explain the negative relationship observed between cell count and distance.

In addition to the influences of spatial constrains on cellular morphology, the proximity of cells to each other, implicated by cell density, may also affect their response to toxicity. Indeed, the influence of cell density on the toxicity of chemicals has been reported, with higher seeding densities being protective of cells from toxicity (Kim and Gilbert 2018; Wu et al. 2020). The differences in BMCs calculated in the four independent experiments could partly be an indication of varying concentration–response caused by differences in cell densities across plates and experimental runs. Similarly, in a multi-laboratory comparison of phenotypic profiles of human hepatocyte cell line Hep G2 exposed to 2464 compounds in 384-well plates, Wolff et al. (2025) noted that the profiles were not readily comparable between different facilities despite high within-site technical replicability (Wolff et al. 2025). Concurrently, the four facilities reported varying average cell counts in the analyzed wells. These differences in cell density—and potentially in response to toxicity—arising from inevitable variations in the number of seeded cells may have contributed to the discrepancies observed across the facilities.

Further work is needed to determine how spatial heterogeneity within and between wells relates to cell count and influences downstream metrics such as Mahalanobis distance. Once this relationship is better defined for U2-OS cells in 96-well plates, developing a correction factor may address the inevitable within-plate and plate-to-plate variabilities in cell density, further refining and improving the consistency of Cell Painting results across different plate formats (Caicedo et al. 2017). Nonetheless, the comparable BMCs generated in this study to those from 384-well plates, as well as the consistent absence of response to two chemicals, NPPB and metoclopramide, suggest that U-2 OS cells responded to the phenotypic reference compounds relatively consistently across both plate formats and studies. Collectively, the current results demonstrate a promising transferability of the assay between formats and laboratories.

Different methods for quantifying concentration–response in Cell Painting data, in addition to the Mahalanobis distance, were explored in Nyffeler et al. (2021). While modeling individual features without dimensionality reduction were sensitive in detecting bioactive chemicals, this method was more prone to producing false positives than the two distance metrics tested, Mahalanobis and Euclidean, with the former having a lower false positive rate (Nyffeler et al. 2021). This method was applied in a more recent study that demonstrated the utility of Cell Painting in chemical hazard screening, which built upon the previous two publications (Nyffeler et al. 2023). Thus, we modeled the Mahalanobis distances to calculate BMCs in our study.

However, with fewer chemicals being exposed on each 96-well plate (three test chemicals in triplicate/plate) compared to a 384-well plate, the Mahalanobis distance metric may not be the most suitable method for quantifying concentration–response relationships in this smaller format. Initially, considering the potential plate-to-plate variability, we normalized each 96-well plate separately rather than treating all plates within an experiment as a single batch. We observed a greater variability in concentrationresponse in the Mahalanobis distance and, consequently, the BMCs for the same chemical across different experiments and within the same experiment (Supplementary Fig. 3). It is possible that 96 samples, compared to 384 samples when 4 plates are batched, may not sufficiently capture the covariance needed for calculating Mahalanobis distances. Similarly, in Nyffeler et al. (2021), all samples across all 48 384-well assay plates were used as input in the analysis, totaling over 15,000 samples in the analysis. In the repeated analysis of the dataset, each 384-well plate was treated independently from the others, and BMCs were calculated from individual plates. Compared to the published BMCs for berberine chloride, etoposide, Ca-074-Me, and rapamycin, there was a larger dispersion of BMCs across the plates in the repeated analysis (Supplementary Fig. 4). This suggests that smaller sample sizes can increase the variability of covariance estimates, as they provide fewer data points to capture the underlying relationships between the variables (Brereton 2015). This increased dispersion in the covariance estimates may result in variable Mahalanobis distance calculations.

The variability within a 96-well plate is inevitably lower than within a 384-well plate. This is important as the variability within the input dataset affects PCA, from which Mahalanobis distances are determined downstream. Small variances may be interpreted as larger distances when the dataset has a lower overall variability (Brereton 2015). Due to this, the chemicals included on a plate may also influence Mahalanobis distance calculation, for example, having negative compounds when running a test screen. Consequently, when there are fewer samples in an experiment (e.g., fewer than four 96-well plates or 384 samples) or the level variability within the dataset is low (e.g., a larger proportion of non-phenotype-altering compounds), the Mahalanobis distance metric may not be the most reliable method for quantifying concentration–response relationships. Further work is needed to determine this variance threshold for deriving reproducible BMCs. In addition, alternative approaches that do not involve feature reduction by PCA and covariance matrix estimation, such as feature or category-level modeling as explored by Nyffeler et al. (2021), may be more suitable for smaller or less variable data such as those from individual 96-well plates. We also recommend continuing to explore other dimensionality reduction techniques that are appropriate for implementation in the cell painting computational pipeline. Additional studies in 96-well plates are required to determine more robust methods for measuring concentration–response for this format.

To support the accessibility of the assay in a medium-throughput laboratory, we opted for a simple plate layout in this study with the three technical replicates in adjacent columns, without positional randomization of chemicals and concentrations within each plate, as done by Nyffeler et al. (2020) using an automated liquid handler. The influence of position in the well on cell viability and response to toxicity, and consequently, the calculated BMCs, was not examined in this study and requires further investigation. Furthermore, the number of technical replicates (wells with the same concentration on a place) also requires additional consideration and optimization. On average, more than 1,200 cells were analyzed per well, with 3 wells treated with the same concentration; roughly over 3,600 cells were analyzed per condition. Reducing the number of technical replicates and incorporating more chemicals per plate (a single well per concentration) may increase the variance across the dataset, which could be advantageous for the PCA-based method, resulting in more reliable Mahalanobis distance calculations. Future studies need to test variations in experimental parameters, such as the position of the solvent control column across plates and the number of technical replicates, to determine the optimal design (e.g., optimal number of cells that provides sufficient statistical power) that would increase the reliability and reproducibility of Cell Painting assay results in 96-well plates, while also considering feasibility in lower throughput settings.

In addition to the experimental and computational challenges, other areas of consideration for utilizing Cell Painting as a chemical screening assay in a medium-throughput laboratory include data storage needs, as discussed by Seal et al. (2025). Each 96-well plate produced approximately 9.5 gigabytes (GB) of images and 1 GB of extracted feature data in a text format. With 10.5 GB of data produced per 96-well plate, this approach could quickly accumulate hundreds of GB of data, making storage and management increasingly challenging. Indeed, data storage is a topic of frequent discussion in bioinformatics dealing with omics data. Options are available that include virtual (e.g., cloud storage) and physical (e.g., hard drives) data storage solutions (Koppad et al. 2021).

All in all, our replication of HTPP results produced in 384-well plates for a set of known phenotype-altering compounds in 96-well plates supports the use of this in vitro chemical screening assay in lower throughput laboratories. Derivation of BMCs from cells exposed in 96-well plates yielded comparable values as exposures and imaging in 384-well plates, demonstrating that HTPP can be performed and produce comparable results across different plate formats. As chemical toxicity testing moves away from in vivo testing, diversification of in vitro endpoints is necessary to obtain biologically relevant information to guide a weight of evidence-based approach for toxicity assessment. The early structural changes in cells detected by HTPP can provide insights into the mechanisms of action to complement other high-throughput toxicity endpoints.

## Supplementary Information

Below is the link to the electronic supplementary material.

Supplementary file1 (DOCX 1772 KB)

## Data Availability

Normalized well-level data are available in the supplementary materials.

## References

[CR1] Ankley GT, Bennett RS, Erickson RJ et al (2009) Adverse outcome pathways: a conceptual framework to support ecotoxicology research and risk assessment. Environ Toxicol Chem 29:730–741. 10.1002/etc.34 10.1002/etc.34 20821501

[CR2] Bray M-A, Singh S, Han H et al (2016) Cell painting, a high-content image-based assay for morphological profiling using multiplexed fluorescent dyes. Nat Protoc 11:1757–1774. 10.1038/nprot.2016.105 27560178 10.1038/nprot.2016.105 PMC5223290

[CR3] Brereton RG (2015) The mahalanobis distance and its relationship to principal component scores. J Chemom 29:143–145. 10.1002/cem.2692

[CR4] Bundy JL, Everett LJ, Rogers JD et al (2024) High-throughput transcriptomics screen of ToxCast chemicals in U-2 OS cells. Toxicol Appl Pharmacol 491:117073. 10.1016/j.taap.2024.117073 39159848 10.1016/j.taap.2024.117073 PMC11626688

[CR5] Caicedo JC, Cooper S, Heigwer F et al (2017) Data-analysis strategies for image-based cell profiling. Nat Methods 14:849–863. 10.1038/nmeth.4397 28858338 10.1038/nmeth.4397 PMC6871000

[CR6] Cimini BA, Chandrasekaran SN, Kost-Alimova M et al (2023) Optimizing the cell painting assay for image-based profiling. Nat Protoc 18:1981–2013. 10.1038/s41596-023-00840-9 37344608 10.1038/s41596-023-00840-9 PMC10536784

[CR7] Culbreth M, Nyffeler J, Willis C et al (2022) Optimization of human neural progenitor cells for an imaging-based high-throughput phenotypic profiling assay for developmental neurotoxicity screening. Front Toxicol. 10.3389/ftox.2021.803987 35295155 10.3389/ftox.2021.803987 PMC8915842

[CR8] Fredin Haslum J, Lardeau C-H, Karlsson J et al (2024) Cell painting-based bioactivity prediction boosts high-throughput screening hit-rates and compound diversity. Nat Commun 15:3470. 10.1038/s41467-024-47171-1 38658534 10.1038/s41467-024-47171-1 PMC11043326

[CR9] Gustafsdottir SM, Ljosa V, Sokolnicki KL et al (2013) Multiplex cytological profiling assay to measure diverse cellular states. PLoS ONE 8:e80999. 10.1371/journal.pone.0080999 24312513 10.1371/journal.pone.0080999 PMC3847047

[CR10] Kavlock RJ, Austin CP, Tice RR (2009) Toxicity testing in the 21st century: implications for human health risk assessment. Risk Anal 29:485–487. 10.1111/j.1539-6924.2008.01168.x 19076321 10.1111/j.1539-6924.2008.01168.x PMC3202604

[CR11] Kim J, Gilbert JL (2018) The effect of cell density, proximity, and time on the cytotoxicity of magnesium and galvanically coupled magnesium–titanium particles *in vitro*. J Biomed Mater Res A 106:1428–1439. 10.1002/jbm.a.36334 29322635 10.1002/jbm.a.36334

[CR12] Koppad S, B A, Gkoutos GV et al (2021) Cloud computing enabled big multi-omics data analytics. Bioinform Biol Insights 15:11779322211035921. 10.1177/11779322211035921 34376975 10.1177/11779322211035921 PMC8323418

[CR13] Krewski D, Andersen ME, Tyshenko MG et al (2020) Toxicity testing in the 21st century: progress in the past decade and future perspectives. Arch Toxicol 94:1–58. 10.1007/s00204-019-02613-4 31848664 10.1007/s00204-019-02613-4

[CR14] Li S, Xia M (2019) Review of high-content screening applications in toxicology. Arch Toxicol 93:3387–3396. 10.1007/s00204-019-02593-5 31664499 10.1007/s00204-019-02593-5 PMC7011178

[CR15] Moffat JG, Rudolph J, Bailey D (2014) Phenotypic screening in cancer drug discovery — past, present and future. Nat Rev Drug Discov 13:588–602. 10.1038/nrd4366 25033736 10.1038/nrd4366

[CR16] Nassiri I, McCall MN (2018) Systematic exploration of cell morphological phenotypes associated with a transcriptomic query. Nucleic Acids Res 46:e116. 10.1093/nar/gky626 30011038 10.1093/nar/gky626 PMC6212779

[CR17] Nyffeler J, Willis C, Lougee R et al (2020) Bioactivity screening of environmental chemicals using imaging-based high-throughput phenotypic profiling. Toxicol Appl Pharmacol 389:114876. 10.1016/j.taap.2019.114876 31899216 10.1016/j.taap.2019.114876 PMC8409064

[CR18] Nyffeler J, Haggard DE, Willis C et al (2021) Comparison of approaches for determining bioactivity hits from high-dimensional profiling data. SLAS Discov 26:292–308. 10.1177/2472555220950245 32862757 10.1177/2472555220950245 PMC8673120

[CR19] Nyffeler J, Willis C, Harris FR et al (2022) Combining phenotypic profiling and targeted RNA-Seq reveals linkages between transcriptional perturbations and chemical effects on cell morphology: retinoic acid as an example. Toxicol Appl Pharmacol 444:116032. 10.1016/j.taap.2022.116032 35483669 10.1016/j.taap.2022.116032 PMC10894461

[CR20] Nyffeler J, Willis C, Harris FR et al (2023) Application of cell painting for chemical hazard evaluation in support of screening-level chemical assessments. Toxicol Appl Pharmacol 468:116513. 10.1016/j.taap.2023.116513 37044265 10.1016/j.taap.2023.116513 PMC11917499

[CR21] R Core Team (2024). R: A language and environment for statistical computing. http://www.R-project.org/.

[CR22] Richard AM, Huang R, Waidyanatha S et al (2021) The Tox21 10k compound library: collaborative chemistry advancing toxicology. Chem Res Toxicol 34:189–216. 10.1021/acs.chemrestox.0c00264 33140634 10.1021/acs.chemrestox.0c00264 PMC7887805

[CR23] Seal S, Trapotsi M-A, Spjuth O et al (2025) Cell painting: a decade of discovery and innovation in cellular imaging. Nat Methods 22:254–268. 10.1038/s41592-024-02528-8 39639168 10.1038/s41592-024-02528-8 PMC11810604

[CR24] Sheffield T, Brown J, Davidson S et al (2022) tcplfit2: an R-language general purpose concentration–response modeling package. Bioinformatics 38:1157–1158. 10.1093/bioinformatics/btab779 34791027 10.1093/bioinformatics/btab779 PMC10202035

[CR25] Svenningsen EB, Poulsen TB (2019) Establishing cell painting in a smaller chemical biology lab – a report from the frontier. Bioorg Med Chem 27:2609–2615. 10.1016/j.bmc.2019.03.052 30935791 10.1016/j.bmc.2019.03.052

[CR26] Swinney DC, Anthony J (2011) How were new medicines discovered? Nat Rev Drug Discov 10:507–519. 10.1038/nrd3480 21701501 10.1038/nrd3480

[CR27] Venables W. N. and Ripley B. D. (2002) Modern Applied Statistics with S. Fourth Edition.

[CR28] Wada K-I, Itoga K, Okano T et al (2011) Hippo pathway regulation by cell morphology and stress fibers. Development 138:3907–3914. 10.1242/dev.070987 21831922 10.1242/dev.070987

[CR29] Way GP, Natoli T, Adeboye A et al (2022) Morphology and gene expression profiling provide complementary information for mapping cell state. Cell Syst 13:911-923.e9. 10.1016/j.cels.2022.10.001 36395727 10.1016/j.cels.2022.10.001 PMC10246468

[CR30] Willis C, Nyffeler J, Harrill J (2020) Phenotypic profiling of reference chemicals across biologically diverse cell types using the cell painting assay. SLAS Discov Adv Sci Drug Discov 25:755–769. 10.1177/2472555220928004 10.1177/2472555220928004 PMC9710725 32546035

[CR31] Wolff C, Neuenschwander M, Beese CJ et al (2025) Morphological profiling data resource enables prediction of chemical compound properties. iScience 28:112445. 10.1016/j.isci.2025.112445 40384930 10.1016/j.isci.2025.112445 PMC12084007

[CR32] Wu Y-K, Tu Y-K, Yu J et al (2020) The influence of cell culture density on the cytotoxicity of adipose-derived stem cells induced by L-ascorbic acid-2-phosphate. Sci Rep 10:104. 10.1038/s41598-019-56875-0 31919399 10.1038/s41598-019-56875-0 PMC6952413

[CR33] Yao K, Rochman ND, Sun SX (2019) Cell type classification and unsupervised morphological phenotyping from low-resolution images using deep learning. Sci Rep 9:13467. 10.1038/s41598-019-50010-9 31530889 10.1038/s41598-019-50010-9 PMC6749053

