# Supplementary material for: Smaller scale, same impact: replicating high-throughput phenotypic profiling in a medium-throughput lab for use in chemical risk assessment
Source: Arch Toxicol. 2025 Aug 28;99(11):4385–97. doi: 10.1007/s00204-025-04165-2 (PMC12477089; doi:10.1007/s00204-025-04165-2)
Supplement: Supplementary file 1 — Supplementary file1 (DOCX 1772 KB) [file 204_2025_4165_MOESM1_ESM.docx]

**Title**: Smaller Scale, Same Impact: Replicating High-Throughput Phenotypic Profiling in a Medium-Throughput Lab for Use in Chemical Risk Assessment

**Supplementary Data**

**Authors**: Eunnara Cho^1^, Stephen D. Baird^2^, Kristin M. Eccles^1^

**Affiliations**:

^1^ Environmental Health Science and Research Bureau, Healthy Environments and Consumer Safety Branch (HECSB) Health Canada, Ottawa, Ontario, Canada

^2^ High-Throughput Screening Lab, Children’s Hospital of Eastern Ontario Research Institute, Ottawa, Ontario, Canada

**Corresponding Author:**

Kristin Eccles

Health Canada

251 Sir Fredrick Banting

Ottawa, ON, K1A 0K9

E-mail: kristin.eccles@hc-sc.gc.ca


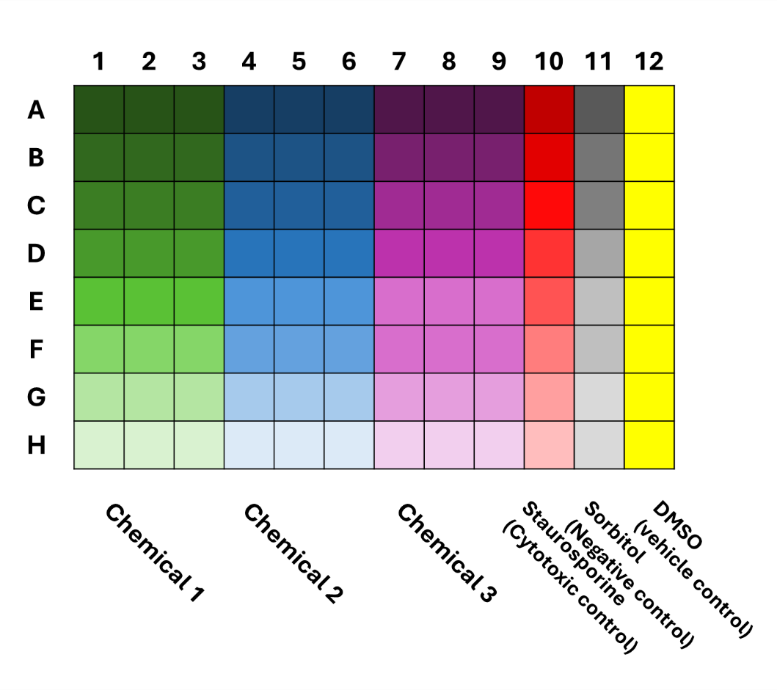


Supplementary Figure 1. Plate layout for chemical exposures and cell painting in a 96-well plate. Three phenotypic chemicals were included in each plate with eight concentrations of staurosporine (cytotoxic control) and sorbitol (negative control) and eight wells treated with the vehicle control (0.5% v/v DMSO). Eight concentrations of each reference compound were included, with the highest concentration in row A and the subsequent concentrations decreasing by half-log unit.


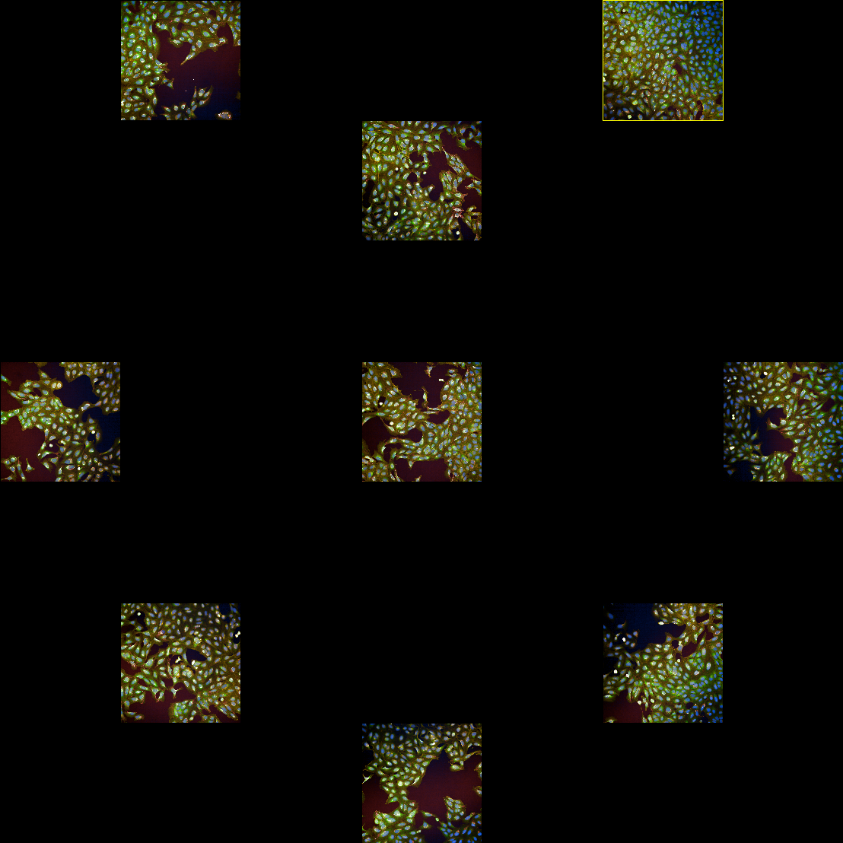


Supplementary Figure 2. Distribution of fields of view across a single DMSO-treated well in a 96-well plate.


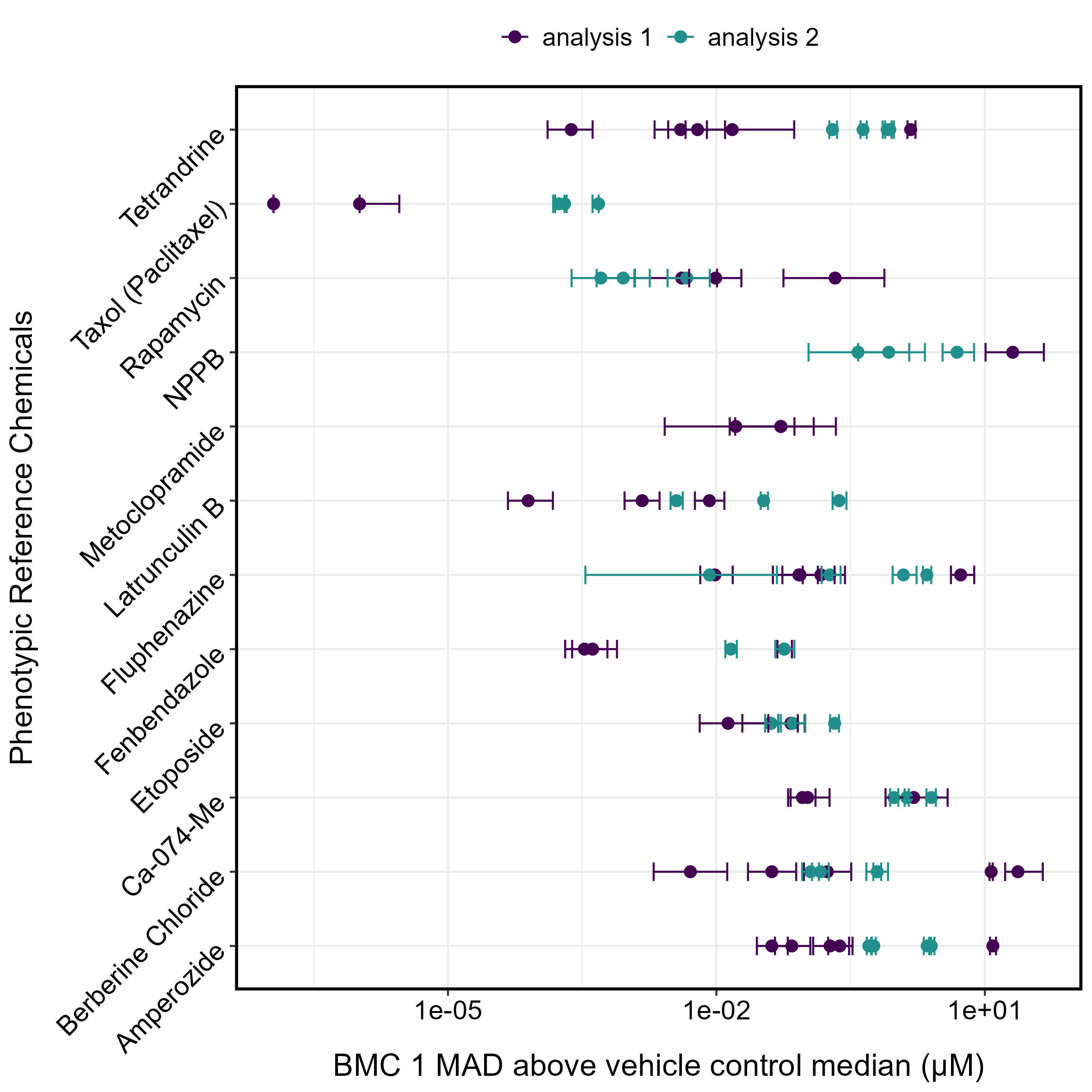


Supplementary Figure 3. Comparison of the benchmark concentrations (BMCs) of the phenotypic reference compounds calculated in the four independent experiments in this study (HC). In analysis 1, each plate was normalized independently to the median and median absolute deviation (MAD) of DMSO-treated cells within the plate. In analysis 2, four plates within each experiment were treated as one 384-sample dataset and were normalized to median and MAD of all DMSO-treated cells across the four plates. The bars represent the 95% confidence intervals (CI) of the BMC values.


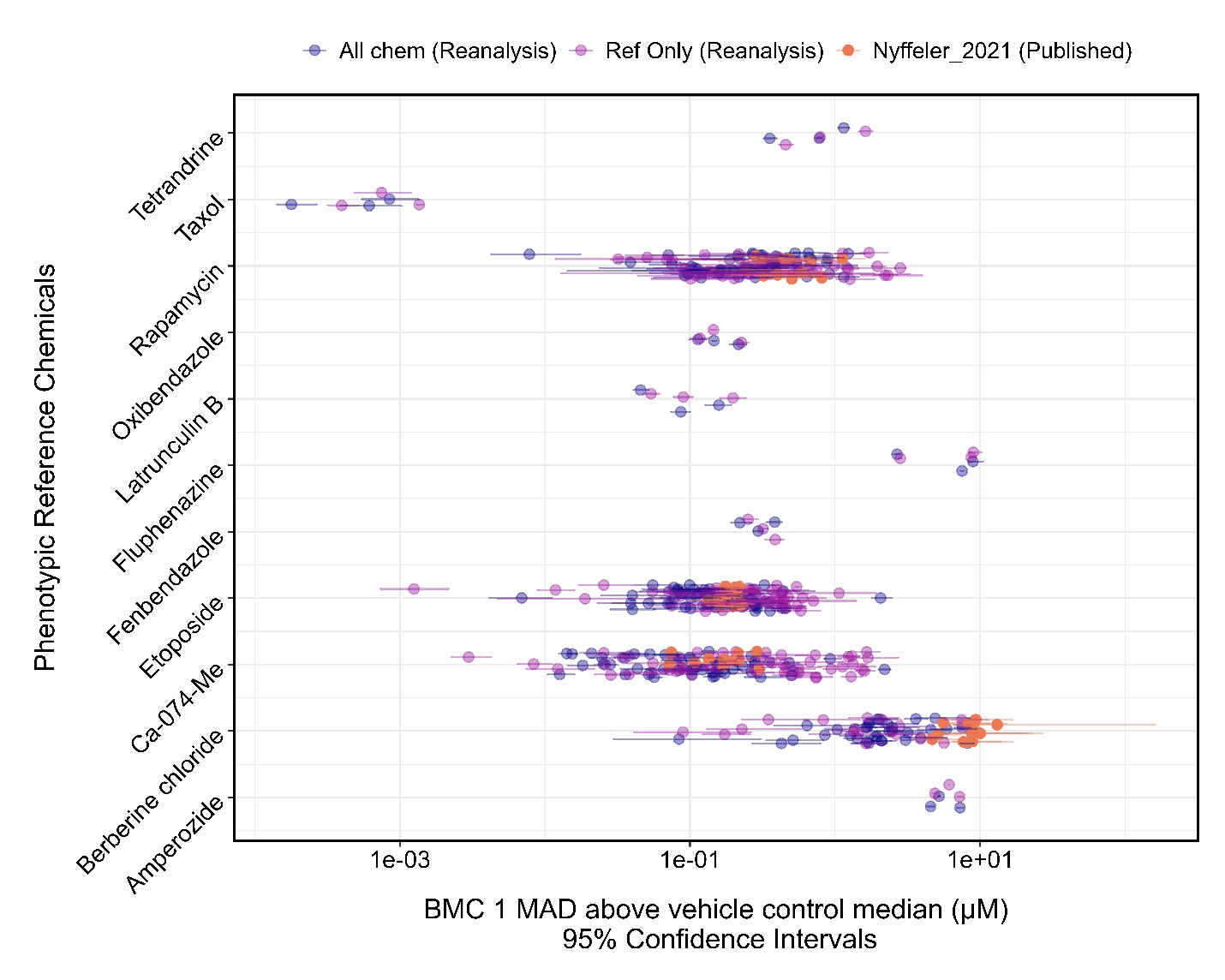


Supplementary Figure 4. Comparison of the benchmark concentrations (BMCs) of the phenotypic reference compounds in the Nyffeler et al. (2020) dataset re-calculated using our modified pipeline. In the “All chem” analysis, one covariance matrix containing all test compounds was generated for each of the 51 plates. In the “Ref Only” analysis, the data for reference compounds were isolated before generating a covariance matrix for each plate. In Nyffeler et al. (2021), 48 384-well plates were divided into 12 groups, each containing four plates from independent cell cultures that were all treated with the same dose plate. Each plate contained rapamycin, etoposide, Ca-074-Me, and berberine chloride as phenotypic positive controls. The bars represent the 95% confidence intervals (CI) of the BMC values.

**
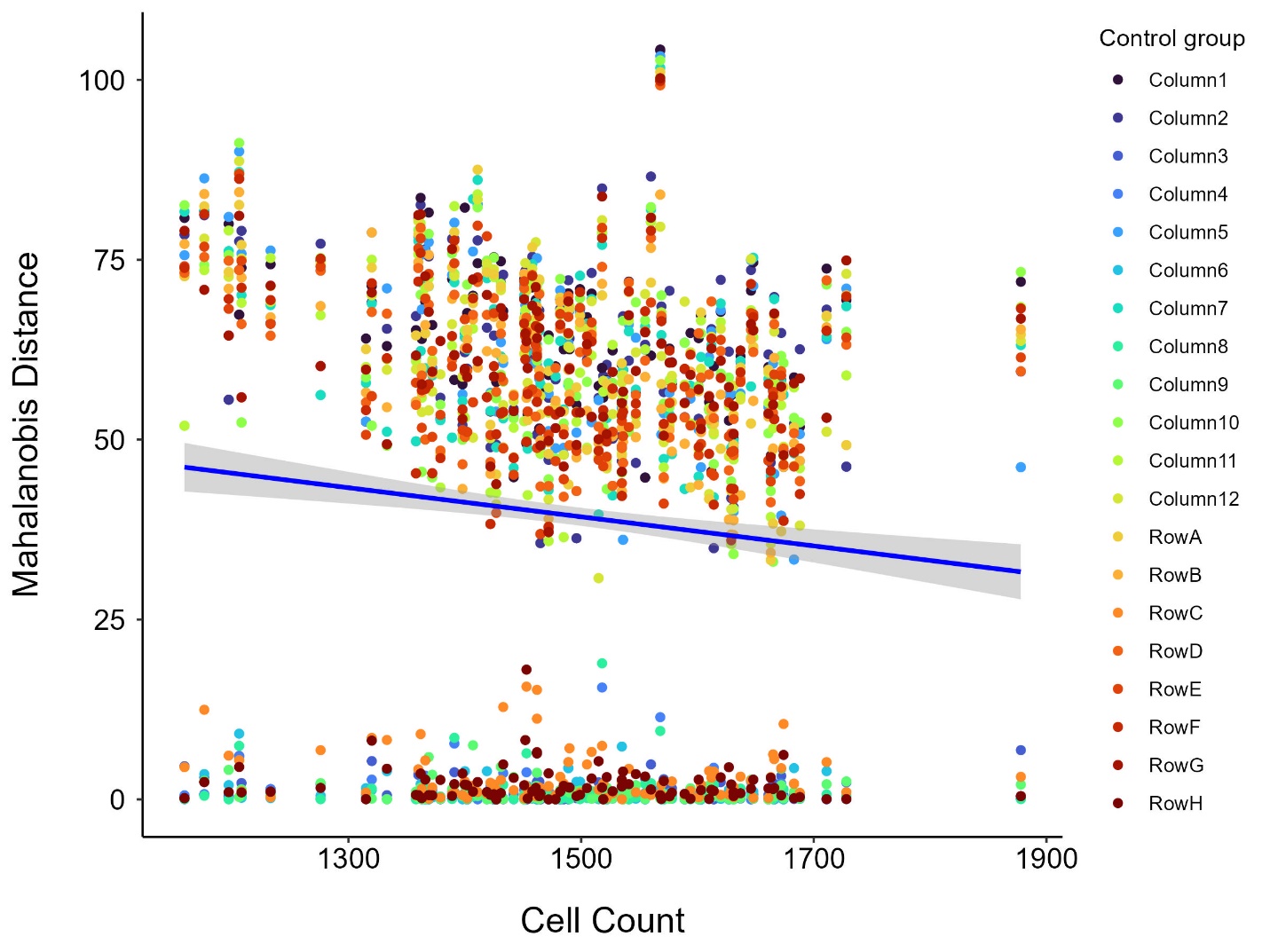
**Supplementary Figure 5. Linear regression of cell count against Mahalanobis distance across different control groups. Control groups correspond to specific plate columns (Column 1–12) or rows (Row A–H). The blue line represents the linear regression fit across all data points, with the shaded area denoting the 95% confidence interval. The adjusted R-squared value indicated that 0.79% of the variance in Mahalanobis distance was attributable to the variance in cell count.
